# Previous gestational diabetes is independently associated with increased carotid intima-media thickness, similarly to metabolic syndrome – a case control study

**DOI:** 10.1186/1475-2840-11-59

**Published:** 2012-05-31

**Authors:** Claudia Maria Vilas Freire, Felipe Batista Lima Barbosa, Maria Cristina C de Almeida, Paulo Augusto Carvalho Miranda, Márcia Melo Barbosa, Anelise Impeliziere Nogueira, Milena Moreira Guimarães, Maria do Carmo Pereira Nunes, Antônio Ribeiro-Oliveira

**Affiliations:** 1 Laboratory of Endocrinology, Department of Internal Medicine, School of Medicine, Federal University of Minas Gerais, Avenida Alfredo Balena 190, Belo Horizonte, MG 30130-100, Brazil; 2 Ecocenter, Socor Hospital, Rua Juiz de Fora 33/3 andar, Belo Horizonte, MG 30180-060, Brazil

**Keywords:** Atherosclerosis, Gestational diabetes, Intima-media thickness, Carotid doppler ultrasonography, Metabolic syndrome

## Abstract

**Background:**

Women with previous gestational diabetes mellitus (pGDM) face a higher risk of developing type 2 diabetes and, consequently, a higher cardiovascular risk. This study aimed to compare the carotid intima-media thickness (cIMT) from young women with pGDM to those with metabolic syndrome (MS) and to healthy controls (CG) to verify whether a past history of pGDM could be independently associated with increased cIMT.

**Methods:**

This is a cross-sectional study performed in two academic referral centers. Seventy-nine women with pGDM, 30 women with MS, and 60 CG aged between 18 and 47 years were enrolled. They all underwent physical examination and had blood glucose, total cholesterol, high-density lipoprotein cholesterol, low-density lipoprotein cholesterol (LDLc), and triglycerides determined. The cIMT was measured by ultrasound in several carotid segments. The primary endpoint was cIMT and clinically relevant parameters included as predictors were: age, systolic blood pressure, waist, BMI, total cholesterol, LDLc, triglycerides, fasting glucose, previous history of GDM as a whole group, previous history of GDM without MS, presence of DM, presence of MS, and parity.

**Results:**

cIMT was significantly higher in pGDM when compared to CG in all sites of measurements (P < 0.05) except for the right common carotid. The pGDM women showed similar cIMT measurements to MS in all sites of measurements, except for the left carotid bifurcation, where it was significantly higher than MS (P < 0.001). In a multivariate analysis which included classical cardiovascular risk factors and was adjusted for confounders, pGDM was shown to be independently associated with increased composite cIMT (P < 0.01). The pGDM without risk factors further showed similar cIMT to MS (P > 0.05) and an increased cIMT when compared to controls (P < 0.05).

**Conclusions:**

Previous GDM was independently associated with increased composite cIMT in this young population, similarly to those with MS and regardless the presence of established cardiovascular risk factors.

## Background

Gestational diabetes mellitus (GDM) is a carbohydrate intolerance detected during pregnancy and its prevalence rate varies between 1-14% [1]. Besides the well-known impact of GDM for the fetus, its effects after pregnancy have been acknowledged [2]. A recent meta-analysis showed a 7.5 times increase in the risk of type 2 diabetes in women with previous GDM and this risk was shown to increase even further when these women were examined more than 5 years postpartum [3]. Besides an increased risk of carbohydrate metabolism disturbances, this population has an increased cardiovascular risk due to clustering of other cardiovascular risk factors such as increased prevalence of metabolic syndrome (MS). However, the presence of known atherosclerotic risk factors may not completely explain the cardiovascular burden in some populations [4,5].

Carotid intima-media thickness (cIMT) measured by ultrasound is an inexpensive test to assess the presence of subclinical atherosclerosis. It assesses the atherosclerotic disease process itself, which includes the net effect of hereditary and environmental factors, either known or yet to be discovered [6]. Moreover, cIMT has been considered an independent predictor of future cardiovascular events such as stroke and coronary artery disease, and it has been used as a surrogate end point in many clinical trials and epidemiological studies [7].

This study aimed to determine whether young women with previous GDM show signs of subclinical atherosclerosis, and whether it could be similar to those with MS. To this purpose, cIMT of pGDM was compared to cIMT of young women with metabolic syndrome (MS) and to young healthy controls.

## Methods

Hundred sixty-nine women were enrolled in this study protocol. Seventy-nine non-smoking and non-menopausal women aged between 18 and 47 years were referred to the Endocrinology Outpatient Clinics of Federal University of Minas Gerais and to Odete Valadares Maternity, 32.51 ± 2.60 months after pregnancies complicated by GDM (pGDM group). They comprised a convenient outpatient sample that had been followed in the outpatient clinics of these both institutions when they had their pregnancies complicated by gestational diabetes. For this pGDM group, any past condition afflicting them at previous pregnancies, other than GDM, was considered an exclusion criteria, especially those requiring hospital admission such as preeclampsia. Thirty non-smoking and non-menopausal patients without previous GDM but with confirmed metabolic syndrome (MS), and 60 healthy non-smoking women within the same age range were enrolled as controls (CG). These two last populations were also derived from the same institutions. Subjects with alcoholism, drug addiction, uremia as well as those with liver, psychiatric, rheumatologic, and thyroid diseases or in use of corticosteroids, were promptly excluded from this protocol.

The diagnosis of GDM was performed as recommended by the American Diabetes Association [8] and risk factors were defined as recommended by the Framingham Study [9], adding body mass index (BMI) and waist [10]. Hypertension was defined according to VII Report of the Joint National Committee on prevention, detection, evaluation and treatment of high blood pressure [11]. For the diagnosis of MS, the definition of the American Heart Association and National Heart Lung and Blood Institute, in which they recommend the Adult Treatment Panel III with minor modifications, was adopted. The combination of three of the following five were used for the diagnosis of MS: waist circumference ≥ 88 cm, triglycerides ≥150 mg/dl or on medication for high triglycerides, high-density lipoprotein cholesterol (HDLc) <50 mg/dl or drug treatment to raise HDLc, blood pressure ≥130/85 mmHg or treatment with antihypertensive medications in a patient with a history of hypertension, and fasting glucose ≥100 mg/dl or drug treatment for hyperglycemia [12].

All participants underwent a thorough physical examination, including the measure of body mass index (BMI) and waist. All medications in use were recorded. They were particularly inquired on their family histories for coronary disease and of current use of hormonal contraceptives. The Framingham’s global cardiovascular risk score (FRS) was estimated to all of them. The risk factors evaluated for the FRS were age, total cholesterol and HDLc, systolic blood pressure, treatment for hypertension, and diabetes status [9].

### Laboratory measurements

Fasting glucose, total cholesterol, HDLc, and triglycerides were all assayed in duplicate. Samples were measured through standard techniques (BIOCLIN, Quibasa, Belo Horizonte, Brazil), and low-density lipoprotein cholesterol (LDLc) was calculated by Friedewald formula as triglycerides was lower than 400mg/dl in all samples.

### Ultrasound study

A GE Health Care Vivid 7 Dimension (Wauwatosa, WI, USA) high-resolution ultrasound scanner with a high frequency (7. 10 or 12 MHZ) linear array transducer was used for cIMT measurement. The examination of the carotid arteries comprised automated measurements of cIMT in selected segments of the far wall: 1cm distal to the flow divider in proximal internal carotid (IC), 1cm proximal to the flow divider as bifurcation (BIF), and 2cm or more proximal to the flow divider in common carotid (CC) bilaterally, as recommended by American Society of Echocardiography [13]. It was measured an average of 243 points for common carotid, and an average of 190 points for internal carotid and bifurcation. The ultrasonographic examination was performed by the same physician (CMVF) blinded to the clinical and metabolic status of the participants, as published elsewhere [14]. The images were checked by another experienced ultrasonographer (MCCA), and the inter-observer coefficient of variation was less than 10%. To quantify for the degree of thickening of the carotid artery walls, the average of the mean cIMT of the three right and three left far wall segments was used to determine the composite cIMT values. As both composite and single measurements have been currently reported in the literature, we have shown both. However, as composite values are representative of the whole measurements, we have chosen the composite approach to the stepwise regression model.

### Statistical analysis

Statistical analysis was performed using SPSS for Windows (Statistical Package for the Social Sciences, Version 17; SPSS, Inc., Chicago, IL, USA). Variables were tested for normality by Shapiro-Wilk test. Continuous data were presented as means ± standard error of the means (SEM) or medians [interquartile ranges], as appropriate. The categorical data were shown as percentage and compared by the chi-square test or the Fisher's exact test whenever the sample sizes were rather small. The clinical, biochemical characteristics and cIMT were compared by Kruskal-Wallis or one-way ANOVA, as appropriate, followed by Bonferroni correction.

We developed a model of multiple linear regression (stepwise selection method, taking P <0.1) using composite cIMT as the dependent variable and clinically relevant parameters as predictors (age, systolic blood pressure, waist, BMI, total cholesterol, LDLc, triglycerides, fasting glucose, previous history of GDM as a whole group, previous history of GDM without MS, presence of DM, presence of MS, and parity). Furthermore, as there was a significant number of women in pGDM group that also had metabolic syndrome, this group was broken down into three (pGDM without risk factor, pGDM with 1 or 2 risk factors, and MS positive) and compared by ANOVA followed by Bonferroni correction to the other groups. A P-value of <0.05 was taken as significant.

### Ethics

All participants signed an informed consent before any procedure related to this study. The institutional Ethical Committees approved the investigational protocol (CAAE – 0455–06).

## Results

The clinical, biochemical, and cIMT characteristics of the studied groups are shown in Tables 1 and 2, respectively. There were no significant differences in age among groups (P > 0.05) Patients from pGDM and MS groups showed significantly higher BMI and waist when compared to CG (P < 0.001 for both comparisons). Likewise, systolic and diastolic blood pressures, fasting glucose, total cholesterol, LDLc, and triglycerides were higher in pGDM and MS when compared to controls (P < 0.001 for all comparisons) whereas HDLc from these two groups was lower than CG (P < 0.001for both comparisons). However, the comparisons between pGDM and MS showed that both BMI and waist were significantly higher in the MS group (P < 0.001 for both comparisons).

**Table 1:** Comparisons of clinical and biochemical characteristics for each group

Variable	pGDM (N = 79)	MS (N = 30)	CG (N = 60)	P
Age (years)	36.42 ± 0.61	37.97 ± 1.14	34.78 ± 0.88	NS
BMI (kg/m^2^)	29.01 ± 0.66	33.32 ± 1.26	22.46 ± 0.42	<0.001^§&^*
Waist (cm)	92.09 ± 1.63	103.63 ± 2.48	74.08 ± 1.14	<0.001^§&^*
Systolic	120.81 ± 1.89	125.67 ± 2.43	112.27 ± 1.02	<0.001^&^*
BP (mmHg)				
Diastolic	79.46 ± 1.30	79.03 ± 1.62	72.63 ± 0.92	<0.01^&^*
BP (mmHg)				
Fasting Glucose (mg/dl)	110.32 ± 5.99	104.96 ± 3.36	84.85 ± 1.06	<0.001^&^*
Total Cholesterol	189.72 ± 5.09	200.32 ± 8.61	171.86 ± 3.74	0.001^&^*
(mg/dl)				
HDLc (mg/dl)	49.71 ± 1.83	45.22 ± 2.17	59.19 ± 1.72	<0.001^&^*
LDLc(mg/dl)	114.21 ± 4.50	123.75 ± 9.39	94.98 ± 3.63	0.001^&^*
Triglycerides	142.05 ± 15.23	177.73 ± 18.50	75.58 ± 3.74	<0.001^&^*
(mg/dl)				

Data are shown as means ± SEM. *pGDM* = previous gestational diabetes group; *MS* = metabolic syndrome group; *CG* = controls; *NS* = not significant; *BMI* = body mass index; *BP* = blood pressure; *HDLc* = high-density lipoprotein cholesterol; *LDLc* = low-density lipoprotein cholesterol; ^&^for comparisons of pGDM to CG; ^§^for comparisons of pGDM to MS; *for comparisons of MS to CG.

**Table 2:** Comparisons of measurements of cIMT for each group of subjects

**cIMT (mm)**	**pGDM group (N = 79)**	**MS Group (N = 30)**	**Control group (N = 60)**	**P**
LCC mean	0.53	0.55	0.50	<0.05^&^
	[0.49-0.58]	[0.46-0.59]	[0.46-0.55]	
LBIF mean	0.61	0.58	0.57	0.001^§&^
	[0.54-0.69]	[0.50-0.64]	[0.50-0.61]	
LIC mean	0.45	0.43	0.40	0.01^&^
	[0.38-0.52]	[0.36-0.50]	[0.32-0.47]	
RCC mean	0.51	0.52	0.50	NS
	[0.46-0.56]	[0.48-0.62]	[0.46-0.54]	
RBIF mean	0.59	0.59	0.55	<0.05^&^*
	[0.51-0.63]	[0.52-0.66]	[0.48-0.60]	
RIC mean	0.45	0.42	0.40	<0.05^&^
	[0.39-0.56]	[0.34-0.52]	[0.36-0.50]	
Composite IMT	0.53	0.53	0.49	<0.001^&^*
	[0.48-0.57]	[0.48-0.57]	[0.45-0.53]	

Data are shown as medians [P25-P75]. *pGDM* = previous gestational diabetes group; *MS* = metabolic syndrome group; *CG* = controls; *NS* = not significant; *R* = right; *L* = left; *CC* = common carotid; *BIF* = bifurcation; *IC* = internal carotid; ^&^ for comparisons of pGDM to CG; ^§^ for comparisons of pGDM to MS; *for comparisons of MS to CG.

Mean cIMT was significantly higher in pGDM when compared to CG in all sites of measurements (P < 0.05 for all measurements), except in the right common carotid where no significant differences could be detected. Interestingly, pGDM and MS showed similar cIMT measures in the left common and in the left internal carotid, besides all right carotid measurements and composite cIMT. However, cIMT was higher in pGDM than MS in the left carotid bifucartion (P < 0.01). MS showed thicker cIMT than controls in the composite (P < 0.001) and right bifurcation measurements (P < 0.05).

Only eight patients from pGDM (10.13%) and four patients from MS (13.33%) were categorized in the intermediate risk category according to FRS (P > 0.05). All patients from CG were categorized as low risk according to FRS. None of the patients were categorized in the high risk group.

**Figure 1:**
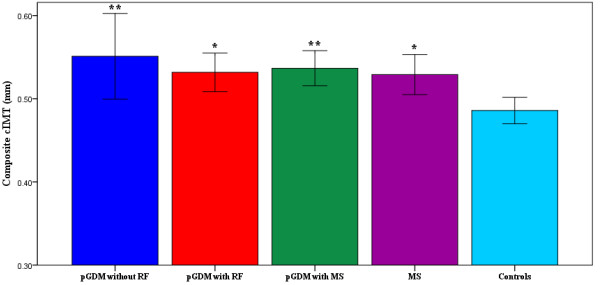
**Graphic representation of the differences in carotid intima-media thickness (means ± SEM) in pGDM without RF (N = 15), pGDM with RF (N = 31), pGDM with MS (N = 33), MS (N = 30), and Controls (N = 60).** cIMT = carotid intima-media thickness; pGDM = previous gestational diabetes group; RF = risk factor; MS = metabolic syndrome * P < 0.05 for comparisons to controls ** P < 0.01 for comparisons to controls.

The pGDM group was a heterogeneous group with respect to the presence of cardiometabolic risk factors. It comprised 15 women with normal cardiovascular profile, 33 women with established MS (three or more features for MS diagnosis) and the others had 1 or 2 risk factors for MS. Therefore, we have divided the pGDM for the purpose of graphic representation of composite cIMT into three subgroups, according to the presence of risk factors or established MS. In Figure 1, we have shown that pGDM without risk factors, pGDM with risk factor(s) and pGDM with MS show higher composite cIMT than controls (0.55 ± 0.02 vs 0.49 ± 0.01, 0.53 ± 0.01 vs 0.49 ± 0.01 and 0.54 ± 0.01 vs 0.49 ± 0.01; P < 0.01, P < 0.05, and P < 0.01, respectively), although these groups did not differ statistically between each other (0.55 ± 0.02 vs 0.53 ± 0.01; 0.53 ± 0.01 vs 0.54 ± 0.01 and 0.55 ± 0.02 vs 0.54 ± 0.01; P > 0.05) and to MS (0.55 ± 0.02 vs 0.53 ± 0.01; 0.53 ± 0.01 vs 0.53 ± 0.01 and 0.54 ± 0.01 vs 0.53 ± 0.01; P > 0.05 for all comparisons). The MS group also showed higher composite cIMT than controls (0.53 ± 0.01vs 0.49 ± 0.01; P < 0.05).

The presence of diabetes, use of hormonal contraception and family history of coronary disease were not statistically different between pGDM and MS groups (P > 0.05 for all comparisons). Specifically, the prevalence of diabetes was 21.52% in pGDM vs 21.43% in MS (P > 0.05). The prevalence of hypertension was significantly lower in pGDM than in MS (30.77% vs 63.33%, respectively, P < 0.01). Antihypertensives were used in 10.11% of pGDM and in 60.01% of MS (P < 0.01), and metformin was used in 12.71% of pGDM and in 13.31% of MS (P > 0.05). Concerning statins, only one patient from pGDM (1.26%) and three patients from MS (10.00%) were using these medications (P > 0.05). None of these patients were on fibrates.

From the predictors taken as possibly related to increased composite cIMT, all of them showed P < 0.1 by the univariate analysis (Table 3). However, only age, total cholesterol, presence of MS and pGDM remained in the multivariate linear regression analysis as independent predictors with P < 0.001, P < 0.01, P < 0.05, and P < 0.01, respectively.

**Table 3:** Predictors of increased composite carotid intima-media thickness from univariate and multivariate stepwise linear regression analysis

**Univariate analysis**			
**Predictors**		**Pearson Correlation**	**P value**
Age (years)		0.503	<0.001
Systolic BP (mmHg)		0.317	<0.001
Waist (cm)		0.308	<0.001
BMI (kg/m^2^)		0.280	0.001
Total cholesterol (mg/dl)		0.329	<0.001
LDLc(mg/dl)		0.311	<0.001
Triglycerides (mg/dl)		0.187	<0.05
Fasting Glucose (mg/dl)		0.196	<0.05
pGDM		0.296	<0.001
pGDM without MS		0.136	>0.05*
Presence of DM		0.139	>0.05*
Presence of MS		0.212	<0.05
Parity		0.281	<0.001
**Multivariate analysis**			
**Predictors**	**Beta**	**(95% CI)**	**P value**
Age (years)	0.431	0.003 – 0.007	<0.001
Total cholesterol (mg/dl)	0.228	0.000 - 0.001	<0.01
MS	0.196	0.007- 0.053	0.01
pGDM	0.210	0.013 – 0.076	<0.01

*BP* = blood pressure; *BMI* = body mass index; *LDLc* = low-density lipoprotein cholesterol; *pGDM* = previous gestational diabetes group; *DM* = diabetes mellitus; *MS* = metabolic syndrome; *CI* = confidence interval. * P < 0,1.

## Discussion

The management of GDM patients after delivery has been considered an important issue to prevent long-term cardiovascular diseases [2]. In the present study, conducted in young women with predominant low FRS, we have demonstrated that pGDM is associated with increased cIMT, a well-established marker of subclinical atherosclerosis, independently of other risk factors commonly observed in this group of women. Women with a past history of GDM had an increased cIMT in most studied carotid segments when compared to controls, but otherwise similar to a group of women with established MS. Interestingly, our pGDM was less hypertensive and obese when compared to our MS. Furthermore, pGDM also showed an increased cIMT in left carotid bifurcation when compared to MS. It has been shown a higher cIMT in the left common carotid than in the right side, although not in internal carotid or carotid bifurcation. These findings may be explained by differences in anatomy and local shear forces, besides bifurcations being an area of predilection for atherosclerotic plaque formation [15,16].

This is the first study, to the best of our knowledge, to show similar cIMT in patients with pGDM as compared to a cohort of patients with MS, shedding new light into this field. However, as our pGDM group was metabolically heterogeneous, it was important to demonstrate that just a past history of GDM is independently associated to increased cIMT regardless of cardiometabolic status and parity. Indeed, the stepwise model here utilized showed the independent predictive value of pGDM for increased cIMT alongside with other strong risk factors for atherosclerotic disease such as age, total cholesterol, and MS [9,10].

Studies using cIMT usually refer to values applied by standard references. In this regard, we have found that the cIMT of our Brazilian pGDM and MS groups were above the 75^th^ percentile when compared to European reference values. However, if we applied the references from North American studies [13], our patients would be classified below the 50^th^ percentile, underestimating their risk. Moreover, due to their young ages, they would probably not be tested for the presence of increased cIMT, especially those without cardiometabolic risk factors. Of note, the increase in cardiometabolic risk factors in this group of young patients with a past history of GDM most often did not result in a change in the FRS towards a higher risk, minimizing possible actions on primary prevention to these women. These data reinforce that it is easy to underestimate the cardiovascular risk in special populations when using the FRS [5,6]. Actually, this study has shown that these young women behave as old from a vascular point of view, although the majority of them were classified as low risk according to FRS.

Several studies have recently shown an increased cardiometabolic risk profile after a pregnancy complicated by GDM, mainly due to abnormalities in glucose homeostasis. Among women with pGDM, the development of postpartum diabetes and metabolic syndrome has been reported as associated with increased cIMT [17]. It is noteworthy that both our pGDM and MS groups show average fasting glucose levels within the range of prediabetes. However, our data show that just a past history of GDM independently account for the increased cIMT. Associated with disorders of glucose metabolism it has been reported an increased prevalence of other risk factors in these women, such as hypertension, obesity and dyslipidemia clustering as MS [3,18]. In addition to these metabolic abnormalities, we have previously shown that women with GDM exhibit alterations on left ventricular diastolic relaxation parameters that do not normalize after delivery, an early stage of diabetic cardiomyopathy [19].

The silent process of atherosclerotic alterations has been imaged by B-mode ultrasound since 1986 [20]. Since then, several studies have shown signs of early changes in the carotid artery wall in populations with classic risk factors, but also in individuals free or well-controlled for them [5,21]. The role of inflammation in atherosclerosis has been well established over the past ten years, and all stages of atherosclerotic plaque formation can be considered as an inflammatory response to injury [22,23]. This relatively new concept that assigns part of the process of development of atherosclerosis to the presence of inflammation could possibly explain the differences in cIMT found between the women from our pGDM group as compared to controls, even when these women were deprived of cardiovascular risk factors. Accordingly, C-reactive protein has been considered a correlate of obesity in women with GDM [24], and the presence of inflammatory markers have been reported in women with pGDM and increased cIMT [25,26].

Recently, Tarim *et al.* showed an increase in left common cIMT in pregnant patients with GDM, independent of fasting glucose, total cholesterol, and HDLc [27]. Volpe *et al.*, studying GDM patients after delivery, reported that these women had higher mean common cIMT, and that levels of oxidized LDL were independently associated with cIMT, thus suggesting an inflammatory component associated with the altered cIMT [25]. Similarly, Bo *et al.* reported that cIMT is significantly associated with pGDM and coupled to a higher prevalence of inflammatory markers [26]. Furthermore, some genetic contribution is also expected to underlie all these findings [4].

The association of IMT with atherogenic dyslipidemia, obesity, hypertension, diabetes and MS has been well demonstrated [7]. However, despite the possible increased risk for atherosclerotic disease with some phenotypes such as MS and obesity, individuals with MS may show signs of subclinical inflammation independently of obesity, while obese people may not present altered metabolic profile [4,28]. Our data showed that this Brazilian cohort of patients with previous GDM show similar cIMT to patients with established MS independently of the cardiometabolic profile, corroborating with an early inflammation condition as an important component in pGDM.

Cross-sectional studies present inherent limitations. Other studies, enrolling larger sample sizes with other ethnical groups, controlling for other confounders such as uric acid [29] and renal function [30] should prospectively contribute to confirm our findings in the future. The present data, however, strengthens the importance of the atherosclerotic process itself in pGDM by direct evaluation of the arterial wall and through comparisons to relevant groups.

The appropriate medical control of our MS group, as observed by the absence of statistical differences in comparisons between pGDM and MS for blood pressure, fasting glucose and lipid levels, despite a higher prevalence of hypertension and obesity in MS, may have contributed to the similarities in cIMT found between these two groups. Another relevant aspect is that the diagnosis of GDM in this study was performed according to *CarpenterCoustan* thresholds [8] while the most current consensus statement has recommended more strict values for this diagnosis [1]. Thus, it is possible that this study has selected women with pGDM who were at higher risk for atherosclerosis thereafter.

## Conclusions

We have shown that just a past history of GDM is associated with an increased cIMT, independently of the presence of risk factors commonly clustered in MS. The demonstration of increased cIMT in this population, similarly to women with altered cardiovascular risk profile, supports an improvement of cardiovascular care of women with gestational diabetes after delivery towards a better cardiovascular outcome.

## Abbreviations

GDM, Gestacional diabetes; IMT, Intima-media thickness; MS, Metabolic syndrome; CG, Control group; BMI, Body mass index; HDLc, High-density lipoprotein cholesterol; LDLc, Low-density lipoprotein cholesterol; FRS, Framingham’s global cardiovascular risk score.

## Competing interests

The authors have no conflict of interest to disclose as related to this study.

## Author’s contributions

CMVF researched data and wrote the manuscript; FBLB researched data; MCCA researched data; PACM researched data and contributed to discussion; MMB reviewed/edited the manuscript; AIN reviewed/edited the manuscript; MMG contributed to discussion and reviewed; MCPN researched data and reviewed the stats; AROJr designed the study and wrote the manuscript. All authors read and approved the final manuscript

## Author’s information

CMVF MD, PhD, cardiologist and cardiovascular ultrasound specialist; FBLB MD; MCCA MD, cardiologist and cardiovascular ultrasound specialist; PACM MD, PhD, endocrinologist; MMB MD, PhD, cardiologist and cardiovascular ultrasound specialist; AIN MD, PhD, endocrinologist and righ risk pregnancy specialist; MMG MD, PhD, endocrinologist; MCPN MD, PhD, cardiologist and cardiovascular ultrasound specialist; AROJr MD, PhD, endocrinologist.

## Funding

This work was supported by Fundação da Pesquisa do Estado de Minas Gerais (FAPEMIG) and Conselho Nacional de Desenvolvimento Científico e Tecnológico (CNPq).
